# Supplementary material for: Identification of photoperiod-induced specific miRNAs in the adrenal glands of Sunite sheep (Ovis aries)
Source: Front Vet Sci. 2022 Jul 22;9:888207. doi: 10.3389/fvets.2022.888207 (PMC9354845; doi:10.3389/fvets.2022.888207)
Supplement: Supplementary file 1 [file Data_Sheet_1.ZIP › Supplementary file/miRDeep2 file.zip!/miRDeep2 file/Know_Structure/SPLP42A2/oar-let-7b.pdf]

miRBase precursor : oar-let-7b
Total read count : 216039
oar-let-7b read count : 215355
remaining reads : 684

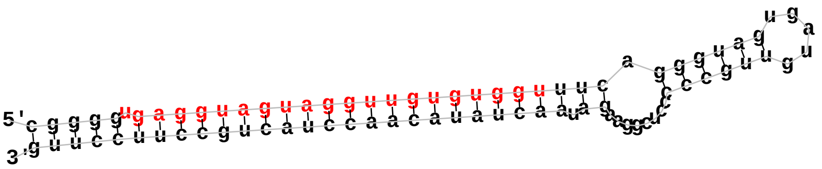

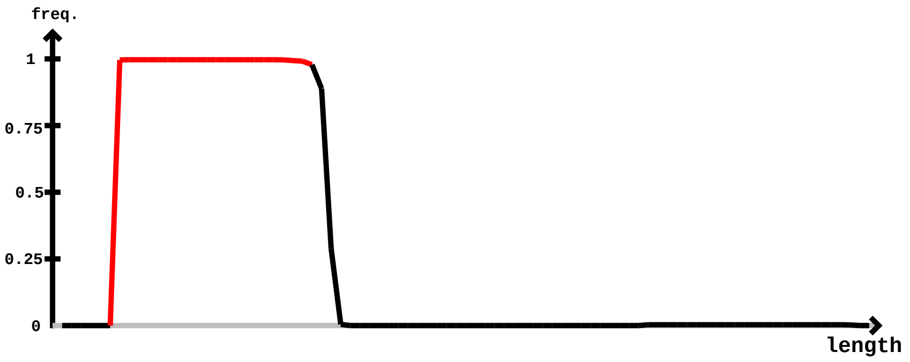


```
        oar-let-7b
5'- cggggugagguaguagguuguguggguuucaggguagugauguugcccccucggaagauaacuauacaaccuacugccuuccuug   -3'   exp
    (((((.(((((((((((((((((((((((.((((((.....))))))(....)..)).))))))))))))))))))))))))))   reads   mm   sample
    ....gugagguaguagguugugu.............................................................   2       0    seq
    ....gugagguaguagguuguguggu..........................................................   1       0    seq
    .....ugagguaguaAguugug..............................................................   1       1    seq
    .....ugagguaguagguugug..............................................................   110     0    seq
    .....ugagguaguagguugugu.............................................................   562     0    seq
    .....ugagguaguaAguugugu.............................................................   2       1    seq
    .....ugagguaguagguugugug............................................................   497     0    seq
    .....ugagguaguagguuguguggg..........................................................   2849    0    seq
    .....ugagguaguagguuguguggu..........................................................   19241   0    seq
    .....ugagguaguagguugugugguu.........................................................   129791  0    seq
    .....ugagguaguagguugugugguuu........................................................   61197   0    seq
    .....ugagguaguagguugugugguuuc.......................................................   933     0    seq
    .....ugagguaguagguugugugguuuca......................................................   5       0    seq
    .....ugagguaguagguugugugguuucagggu..................................................   1       0    seq
    .....ugagguaguagguugugugguuucaggguagug..............................................   1       0    seq
    ......gagguaguagguugugugg...........................................................   2       0    seq
    ......gagguaguagguuguguggu..........................................................   9       0    seq
    ......gagguaguagguugugugguu.........................................................   90      0    seq
    ......gagguaguagguugugugguuu........................................................   57      0    seq
    .......agguaguagguugugugguu.........................................................   1       0    seq
    .......agguaguagguugugugguuu........................................................   3       0    seq
    ........gguaguagguugugugguu.........................................................   1       0    seq
    ..........uaguagguugugugguu.........................................................   1       0    seq
    ..........uaguagguugugugguuucaggg...................................................   1       0    seq
    ...........................ucaggguagugauguugcccccucggaa.............................   1       0    seq
    .............................................ccccucggaagauaacuauacaac...............   1       0    seq
    ...............................................ccucggaagauaacuauacaaccu.............   1       0    seq
    ......................................................agauaacuauacaaccuacug.........   1       0    seq
    .......................................................gauaacuauacaaccuacugccu......   1       0    seq
    .........................................................uaacuauacaaccuacugccuu.....   1       0    seq
    .........................................................uaacuauacaaccuacugccuucc...   1       0    seq
    ..........................................................aacuauacaaccuacugccuucc...   1       0    seq
    ..........................................................aacuauacaaccuacugccuuccu..   1       0    seq
    ............................................................cuauacaaccuacugccuu.....   1       0    seq
    ............................................................cuauacaaccuacugccuuc....   11      0    seq
```

oar-let-7b

```
cggggugagguaguagguuguguggu uucaggguagugauguugcccccucggaagauaacuauacaaccuacugccuuccuug
```

cggggugagguaguagguugugugguuucaggguagugauguugcccccucggaagauaacuauacaaccuacugccuuccuug			
....cuauacaaccuacugccuucc...	121	0	seq
....cuauacaaccuacugccuuccu..	510	0	seq
....cuauacaaccuacugccuuccuu.	19	0	seq
....cuauacaaccuacugccuuccuug	1	0	seq
....uauacaaccuacugccuuccu..	6	0	seq
....auacaaccuacugccuuccu..	3	0	seq
